# Karyomorphometric analysis of *Fritillaria montana* group in Greece

**DOI:** 10.3897/CompCytogen.v10i4.10156

**Published:** 2016-12-01

**Authors:** Sofia Samaropoulou, Pepy Bareka, Georgia Kamari

**Affiliations:** 1 Laboratory of Systematic Botany, Faculty of Crop Science, Agricultural University of Athens, Iera Odos 75, 118 55 Athens, Greece; 2 Botanical Institute, Section of Plant Biology, Department of Biology, University of Patras, 265 00 Patras, Greece

**Keywords:** Fritillaria epirotica, Fritillaria montana, karyotype analysis, PCoA, endemics, Greek flora, karyograms

## Abstract

*Fritillaria* Linnaeus, 1753 (Liliaceae) is a genus of geophytes, represented in Greece by 29 taxa. Most of the Greek species are endemic to the country and/or threatened. Although their classical cytotaxonomic studies have already been presented, no karyomorphometric analysis has ever been given. In the present study, the cytological results of *Fritillaria montana* Hoppe ex W.D.J. Koch, 1832 group, which includes *Fritillaria epirotica* Turrill ex Rix, 1975 and *Fritillaria montana* are statistically evaluated for the first time. Further indices about interchromosomal and intrachromosomal asymmetry are given. A new population of *Fritillaria epirotica* is also investigated, while for *Fritillaria montana*, a diploid individual was found in a known as triploid population. Paired t-tests and PCoA analysis have been applied to compare the two species.

## Introduction

The genus *Fritillaria* Linnaeus, 1753 (Liliaceae) comprises approximately 150 taxa of geophytes, found in the temperate zones of the Northern Hemisphere (Kamari and Phitos 2006). Most of them are distributed across Eurasia while about 20 species occur in California. Only one species, *Fritillaria camschatcensis* (L.) Ker Gawler, 1809 links both groups by its distribution in both North America and eastern Asia (Fay and Chase 2000, Ambrozova et al. 2011).

According to its latest revision (Rix 2001), the genus is divided into eight subgenera, *Fritillaria* Rix, 2001 (including two sections, *Olostyleae* Rix, 2001 and *Fritillaria* Rix, 2001); *Davidii* Rix, 2001; *Liliorhiza* (Kellogg) Bentham & Hooker, 1883; *Japonica* Rix, 2001; *Rhinopetalum* Fischer, 1835; *Petilium* Baker, 1874; the monotypic *Theresia* K. Koch, 1849 and *Korolkowia* Rix, 2001. Although Iran (and more precisely its northern part as well as the neighbouring countries) is relatively poor in species (17 species and 4 subspecies), it is considered to be the centre of *Fritillaria* diversity above species level (Rix 1977), because those taxa belong to four out of five main subgenera (Jafari et al. 2014).

In Greece, the genus is also characterized by high diversity and is represented by a multitude of taxa (24 species and 5 subspecies), all belonging to the subgenus Fritillaria (Kamari and Phitos 2006).

Out of the 29 taxa found or described in Greece so far, 18 taxa (14 species and 4 subspecies) are endemic to the country and no less than 17 species and 2 subspecies occur in the Aegean archipelago and the surrounding continental region (Kamari and Phitos 2000). Moreover, Turkey is the richest country concerning the number of *Fritillaria* with 35 species and 6 subspecies, 19 of which are considered endemic (Tekşen and Aytaç 2011, Advay et al. 2015, Özhatay et al. 2015). Eighteen of those species and 4 subspecies are distributed in the Mediterranean, 12 of which are endemic. Taking into consideration the total number of *Fritillaria* taxa as well as the number of the endemic ones, Greece, along with W Turkey (Rix 1984, Özhatay 2000, Tekşen 2012), constitutes a secondary evolutionary center at least for this subgenus, if not for the whole genus. As a result, the Aegean archipelago can be considered as the heart of the secondary biodiversity center for the subgenus Fritillaria (Kamari and Phitos 2000).

Among the *Fritillaria* taxa occurring in continental Greece two species constitute the *Fritillaria montana* group (Kamari 1991a): *Fritillaria epirotica* Turrill ex Rix, 1975, which is endemic to NW Greece and *Fritillaria montana* Hoppe ex W.D.J.Koch, 1832, which has a wide distribution in S and SE Europe. Both species of the above group are characterized by their long (2/3 of the tepal length) nectaries, as well as by their obscurely tessellated tepals.

*Fritillaria epirotica* is a very short plant (up to 15 cm) with dark purplish, obscurely tessellated flowers, which almost touch the ground and it grows on ophiolithic substrates, usually at high altitudes (up to 2600 m). On the contrary, *Fritillaria montana* is tall (up to 60 cm), characterized by alternate or subopposite linear, slightly canaliculated leaves, with dark purplish distinctly tessellated flowers, and it grows usually on limestone substrate at an altitude up to 1600 m.

*Fritillaria epirotica* is included in the Red Data Book of Rare and Threatened Plants of Greece (Phitos et al. 1995 & 2009), in the IUCN Red List of Threatened Species, Version 2014.2. and also in the Council Directive 92/43/EEC on the conservation of natural habitats and of wild fauna and flora. It is protected by the Presidential Decree 67/81, characterized as Endangered (EN) by IUCN and as Vulnerable (VU) in the Red Data Book of Rare and Threatened Plants of Greece (Kamari 1995, Kamari and Phitos 2009). *Fritillaria montana* is characterized, according to IUCN Red List of Threatened Species, Version 2014.2., as a Data Deficient (DD) species. In Greece, some of its populations are included in Natura 2000 sites. Despite its wide distribution, the species is Rare (R) in Italy (Peruzzi et al. 2008) and included in the regional Red Lists of Italian threatened species (Conti et al. 1997). As already known, the misapplied nomenclature of the *Fritillaria montana* complicates botanical literature (Lozina-Lozinskaja 1935, Zahariadi 1966, Kamari 1991a, b, Tomovic et al. 2007). Several locations in Italy have recently been further studied and *Fritillaria montana* populations are getting distinguished, while more biometric details for the species are provided (Peruzzi and Bartolucci 2009, Bartolucci et al. 2009, Mancuso et al. 2012, Peruzzi et al. 2012). An indicative example of the situation is the very low production of fruits during fruiting season in 2008 observed by Mancuso et al. (2012). Moreover, *Fritillaria montana* is characterized as an Endangered (EN) species listed in the third edition of the Red Book of Ukraine (Chorney et al. 2009), as a Rare (R) one in Bosnia and Herzegovina (Šilić 1996), Vulnerable (VU) in Serbia (IUCN Red List of Threatened Species) and protected at a national level in France. Tomovic et al. (2007) referred that the species was listed as Rare in the Red Data Book of the PR Bulgaria (Velchev 1984 sub *Fritillaria orientalis* Adam), but the latest version does not include it anymore (Petrova and Vladimirov 2009).

Concerning the cytology of the genus, *Fritillaria* has been studied for many years due to the interest of its large chromosomes and vast genome size (Darlington 1935, 1937, Frankel 1940). Indeed, 1C values (DNA content of the unreplicated haploid chromosome complement) in *Fritillaria* are among the largest reported for all angiosperms (Bennett and Smith 1976, Sharma and Raina 2005). The karyotype is quite stable, asymmetrical and usually diploid, with a basic chromosome number of x = 12. Only a few species are an exception to this, with x = 9 (3 species), x = 11 (2 species) and x = 13 (2 species), but without a special pattern (Darlington 1937, Noda 1964, Li and Shang 1989, Jafari et al. 2014). However, the presence and the morphology of satellited chromosomes vary among the species or even among populations of the same taxon (Runemark 1970, Bentzer et al. 1971, Mehra and Sachdeva 1976, Koul and Wafai 1980, Kamari 1984a, 1991a, 1996, Zaharof 1987, Kamari and Phitos 2006). In addition, secondary constrictions and supernumerary B-chromosomes are observed very often (La Cour 1978b, 1978c, Kamari 1984a, 1991a, 1991b, Zaharof 1987, 1989, Kamari and Phitos 2006). As a result, that type of differentiations is always emphasized and specific chromosome pairs are studied as markers, in order to spot the differences among the generally stable and similar karyotypes (Kamari 1984b, Zaharof 1989, Kamari and Phitos 2000, 2006). Finally, a few triploid karyotypes have been reported with 2n = 3x = 36 (Fedorov 1969, La Cour 1978a, Moore 1982, Marchant and Macfarlane 1980, Zaharof 1987, Peruzzi et al. 2009) or with 2n = 3x = 27 chromosomes (Cesca 1986, Kamari 1991a).

Recently many questions have arisen, regarding the classification and phylogeny of the genus, especially for the species appearing in Greece. Although several molecular phylogenetic studies have been published (Fay and Chase 2000, Rønsted et al. 2005, Turktas et al. 2012, Metin et al. 2013) none of them refer to the total of Greek taxa. Even though classical cytotaxonomic studies of the genus in Greece have already been published (Kamari 1984a, 1984b, 1991a, 1991b, 1996, Zaharof 1987, 1989, Kamari and Phitos 2000, 2006), neither karyomorphometric analysis, nor statistical evaluation of the cytological results, have ever been given so far. In the present study, an attempt for further karyomorphometric analysis of chromosome features has been made, concerning the two members of *Fritillaria montana* group.

## Material and methods

Living plants of the *Fritillaria montana* and *Fritillaria epirotica* populations were collected (Table 1) and cultivated in the Experimental Gardens of the University of Patras and Agricultural University of Athens. Vouchers are deposited in UPA and ACA.

**Table 1.** Origin, chromosome numbers (2n) and voucher number of *Fritillaria* material.

Taxon	Origin	2n	Voucher number, Herbarium
*Fritillaria montana*	Mt. Vourinos (W Macedonia)	18	16765, UPA
	Mt. Kato Olympos (Thessalia)	18	SF1089, ACA cult. no. 253, UPA
	Mt. Boutsi (NW Macedonia)	27 and 18 (1 individual)	SF1092, ACA 19865, UPA
*Fritillaria epirotica*	Katara Pass (Epirus)	24	21348, UPA 7919, UPA
	Mt. Vasilitsa (N Pindos)	24	SF1076, ACA
	Mt. Smolikas (N Pindos)	24	SF1097, ACA
	Mt. Kratsovo (W Thessalia)	24	cult. no. 255, UPA

The cytological study is based on the squash technique and the chromosome counts were made from root tip metaphases (Östergren and Heneen 1962, Kamari 1976). The root tips were pretreated in a mixture of 1:1 8-hydroxyquinoline (0,002% w/v):colchicine (0.3 w/v) for 3 hrs (Kamari 1984a) and fixed in 3:1 (v/v) absolute ethanol:glacial acetic acid for 24 hours at 4 °C. Fixed root tips were stored at -20 °C at 75% ethanol.

Before staining, the root tips were hydrolyzed in 1N HCl 60 °C for 15 min and stained in Feulgen for 3 hrs (Darlington and La Cour 1969). Prior to squashing, the stained root tips were put on a slide with a drop of 45% (v/v) acetic acid. The slides were observed with AXIOLAB Zeiss microscope and photos were taken with Canon EOS 600D digital camera.

At least five metaphase plates of each species were analysed and indices were calculated with Microsoft Office Excel 2007, IBM SPSS Statistics version 22 and Past 3.03. Chromosome terminology follows Levan et al. (1965), Stebbins (1971) and Kamari (1976), taking into consideration comments and suggestions by Sybenga (1959), Bentzer et al. (1971) and Favarger (1978). For each taxon there is a presentation of the karyotype formula, maximum and minimum length of the chromosomes, total and average chromosome length and total haploid length of the chromosome set, along with their standard deviation. The interchromosomal asymmetry (CV_CL_), is estimated according to Paszko (2006) and the intrachromosomal asymmetry (M_CA_) according to Watanabe et al. (1999), Peruzzi and Eroğlu (2013) and Peruzzi and Altinordu (2014). Additionally the coefficient of variation of centromeric index (CV_CI_) measuring the centromere position heterogeneity is estimated following Paszko (2006) and Peruzzi and Altinordu (2014). A multivariate analysis (Principal Coordinate Analysis - PCoA) was made concerning six karyological parameters: 2n, x, THL, CV_CL_, CV_CI_ and M_CA_ (Peruzzi and Altinordu 2014). When marker chromosomes are observed (metacentric, submetacentric, SAT-chromosomes and secondary constrictions) r-index, R-length, Centromeric index and Arm difference ratio are also given. Finally, t-tests are given, regarding the indices of TCL, ACL, CV_CL_, M_CA,_ in order to check statistically significant differences between the two species.

## Results

**Figure 1.**
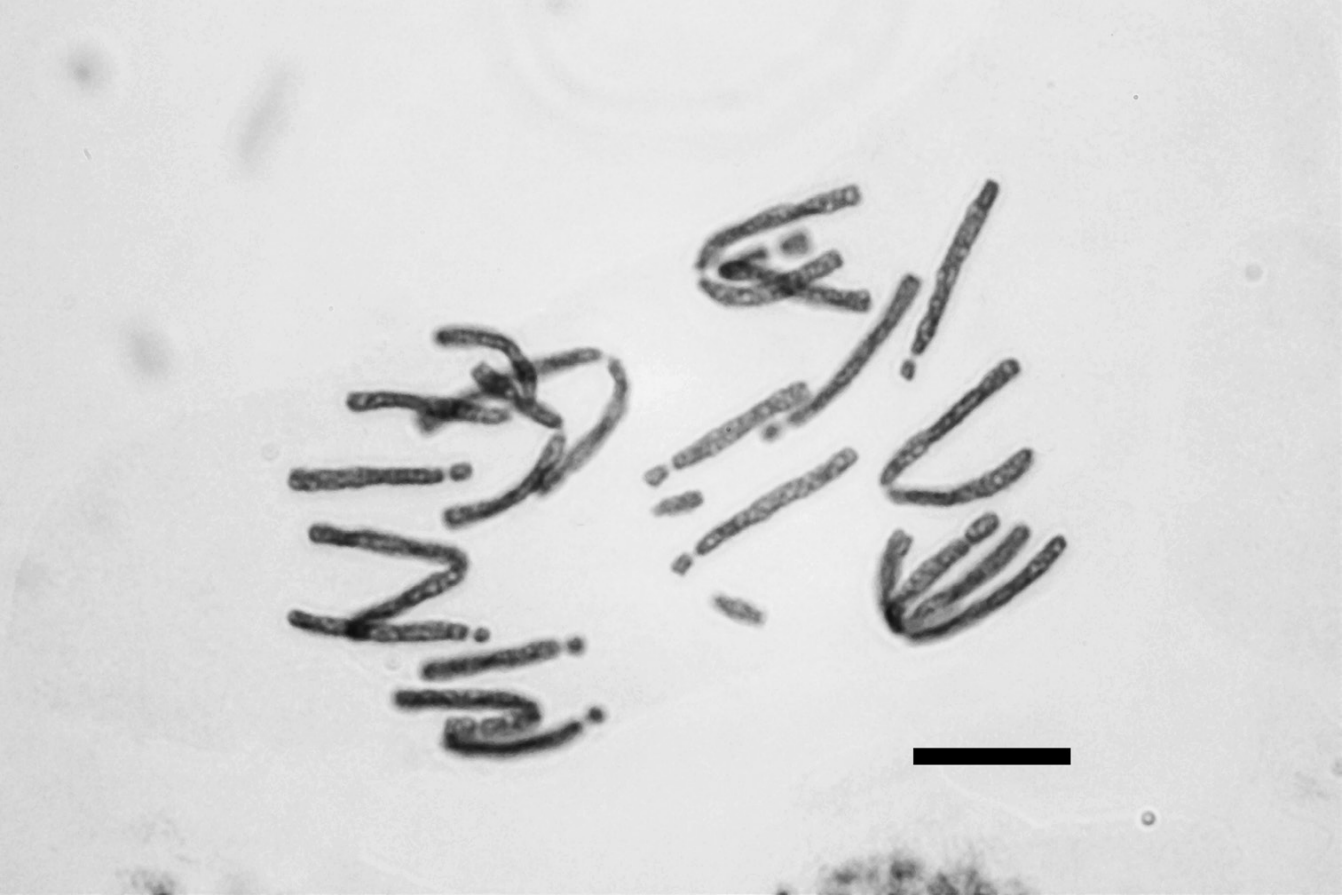
Photomicrograph of mitotic metaphase plate of *Fritillaria montana* from Mt. Vourinos, 2n = 2x = 18. Bar = 10 µm.

**Figure 2.**
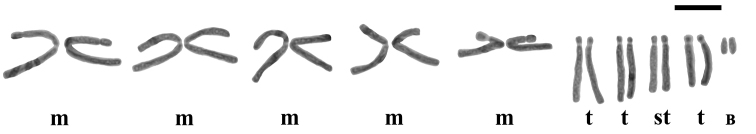
Karyogram of *Fritillaria montana* from Mt. Vourinos, 2n = 2x = 18. Bar = 10 µm.

***Fritillaria montana*** Hoppe — 2n = 2x = 18 + 0-3B (Figs 1, 2).

Populations karyologically studied:

**Greece: Macedonia: Nomos Kozanis**: mons Vourinos, in declivibus orientalibus cacuminis, alt. 1300-1350 m, in apertis ad viam et in silva Abietis et Pinetis, solo ophiolithico, 9 Jul 1981, T.R. Dudley, D. Phitos, D. Tzanoudakis, Gr. Iatrou & D. Christodoulakis 16765 (UPA); **Thessalia: Nomos Larissis**: Mt. Kato Olympos, Livadaki, north of Kallipefki, alt. ca. 1407 m, 39°57'N; 22°29'E, 30 May 2015, S. Samaropoulou, I. Patrikios & K. Tamvakas SF1089 (ACA); Mt. Kato Olympos, Livadaki, alt. 1400 m, May 2006, K. Tamvakas 253 (UPA).

*Fritillaria montana* is the only Greek species with a basic chromosome number of x = 9, having 2n = 18 chromosomes (Fig. 1). Its karyotype includes two metacentric (m) chromosome pairs that can be characterized as markers, the longer and the shorter ones (Table 3, chromosome pairs no. 1 and no. 5, numbered according to their chromosome length), because they bear characteristic secondary constrictions close to the end of the short arm (Fig. 2). Secondary constrictions are also observed to the rest of the metacetric chromosomes, however, they are not always visible. For this reason, the other three metacentric chromosome pairs cannot be characterized as markers.

The karyotype formula of the studied populations is given as 2n = 10m + 2st + 6t = 18 (Fig. 2). The chromosome size ranges between 24.41 µm and 11.26 µm and the total chromosome length is 316.34 µm. The karyotype is more symmetric (Table 2) concerning the variation in chromosome length (CV_CL_ = 25.2) rather than the centromere position (M_CA_ = 41.42), while the parameter CV_CI_ is even higher (CV_CI_ = 56.21). Up to three B-chromosomes were found, all of them acrocentric (st) in the studied material.

**Table 2.** Studied species with karyomorphometric indices. Chromosome number (2n), total (TCL) and average (ACL) chromosome length, total haploid chromosome length (THL), maximum (max l + s) and minimum (min l + s) chromosome length, karyotype asymmetry indices (CV_CL_, CV_CI_ and M_CA_).

Species	*Fritillaria montana*		*Fritillaria epirotica*
**Chromosome number**	2n = 2x = 18	2n = 3x = 27	2n = 2x = 24
**Karyotype formula**	10m + 2st + 6t	15m + 3st + 9t (10m + 4st + 4t, 1 individual)	2m + 2sm + 14st + 6t
**TCL (µm)** **(SD)**	316.34 (30.22)	363.23 (53.47)	324.39 (51.12)
**THL (µm)** **(SD)**	158.17 (15.11)	121.08 (17.82)	162.2 (25.56)
**ACL (µm)** **(SD)**	17.57 (1.68)	13.45 (1.98)	13.52 (2.13)
**max l + s (µm)**	24.41	22.86	18.44
**min l + s (µm)**	11.26	8.00	10.00
**CV_CL_** **(SD)**	25.26 (1.12)	31.07 (3.61)	16.85 (2.43)
**CV_CI_** **(SD)**	56.21 (0.99)	54.79 (3.57)	51.66 (2.99)
**M_CA_** **(SD)**	41.42 (0.35)	40.41 (1.18)	63.33 (1.25)

**Figure 3.**
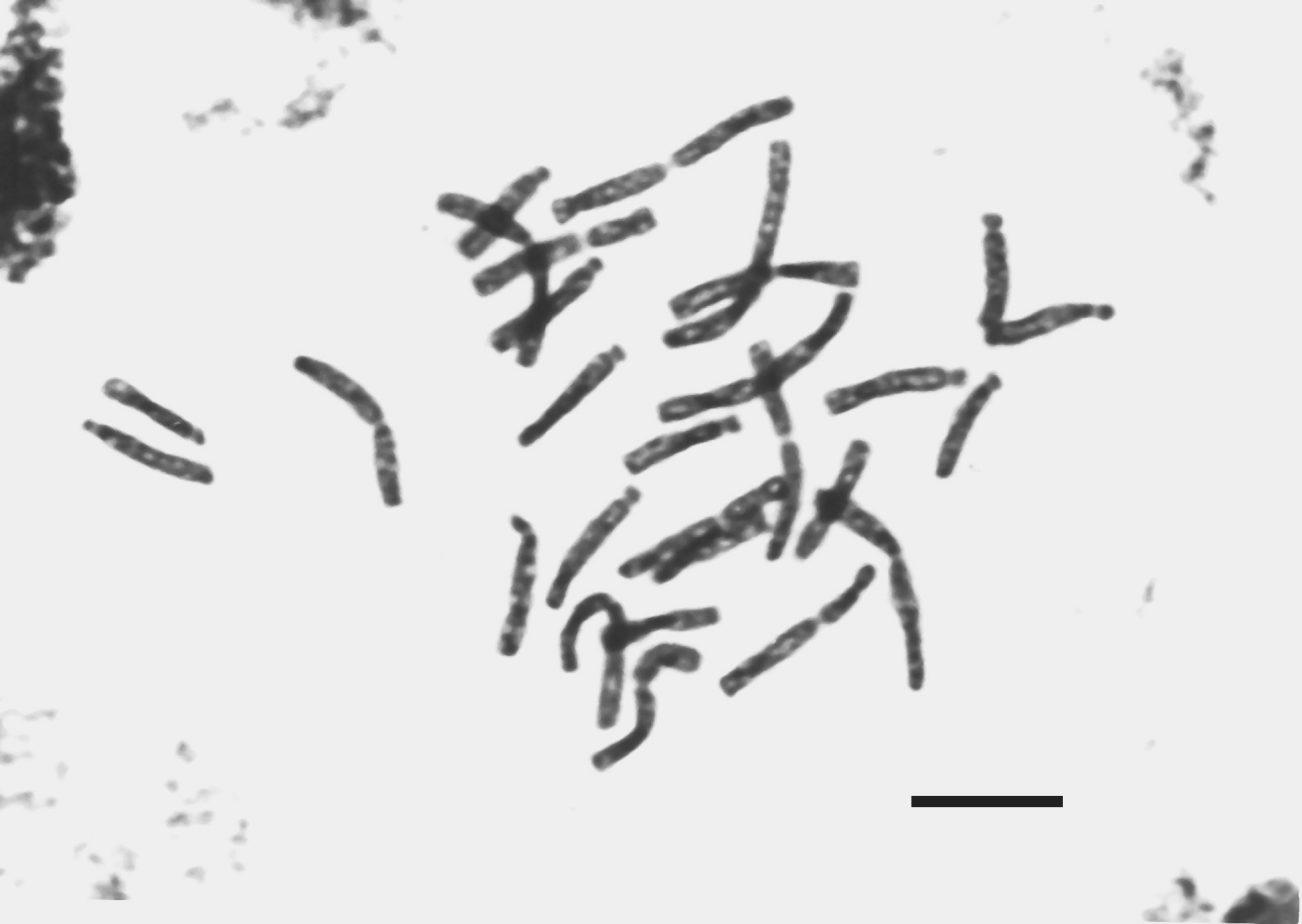
Photomicrograph of mitotic metaphase plate of *Fritillaria montana* from Mt. Boutsi, 2n = 3x = 27. Bar = 10 µm.

**Figure 4.**
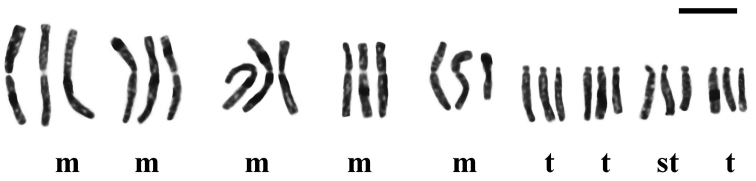
Karyogram of *Fritillaria montana* from Mt. Boutsi, 2n = 3x = 27. Bar = 10 µm.

***Fritillaria montana*** Hoppe — 2n = 3x = 27 and 2n = 2x = 18 (1 individual) (Figs 3, 4).

Populations karyologically studied:

**Greece: Macedonia: Nomos Florinas**: Montes Triklarion, in declivibus boreo-occidentalibus cacuminis Boutsi, in apertis saxosis calc., alt. 1450-1550 m, 19 May 1987, D. Phitos & G. Kamari 19865 (UPA); Mt. Boutsi, alpine meadow, calcareous substrate, alt. ca. 1549 m, 40°38'33"N; 21°09'25"E, 2 Jun 2015, S. Samaropoulou, I. Patrikios & A. Ioannou, sub Samaropoulou SF1092 (ACA).

The triploid population previously reported for the first time by Kamari (1991a), is now further examined. The karyotype formula is given as 2n = 15m + 3st + 9t = 27 (Figs 3, 4) and the chromosome length ranges from 22.86 µm to 8 µm, while the TCL equals to 363.23 µm (Table 2). The interchromosomal asymmetry of the triploid karyotype (CV_CL _= 31.07) is higher than the diploid, but the intrachromosomal is lower (M_CA_ = 40.41). The heterogeneity of the centromere position is lower than the diploid (CV_CI_ = 54.79). Even though secondary constrictions were observed again, their number and position varies in several plates, making the distinction of marker chromosomes very difficult.

It is noteworthy that a diploid individual was found for the first time at the studied triploid population. The karyotype of this individual comprises 2n = 10m + 4st + 4t = 18 chromosomes, with an additional pair of acrocentric (st) chromosomes compared with the other diploid populations studied and without B-chromosomes in contrast with the population of Mt. Vourinos. The secondary constrictions were also unclear as in the triploid individuals.

**Figure 5.**
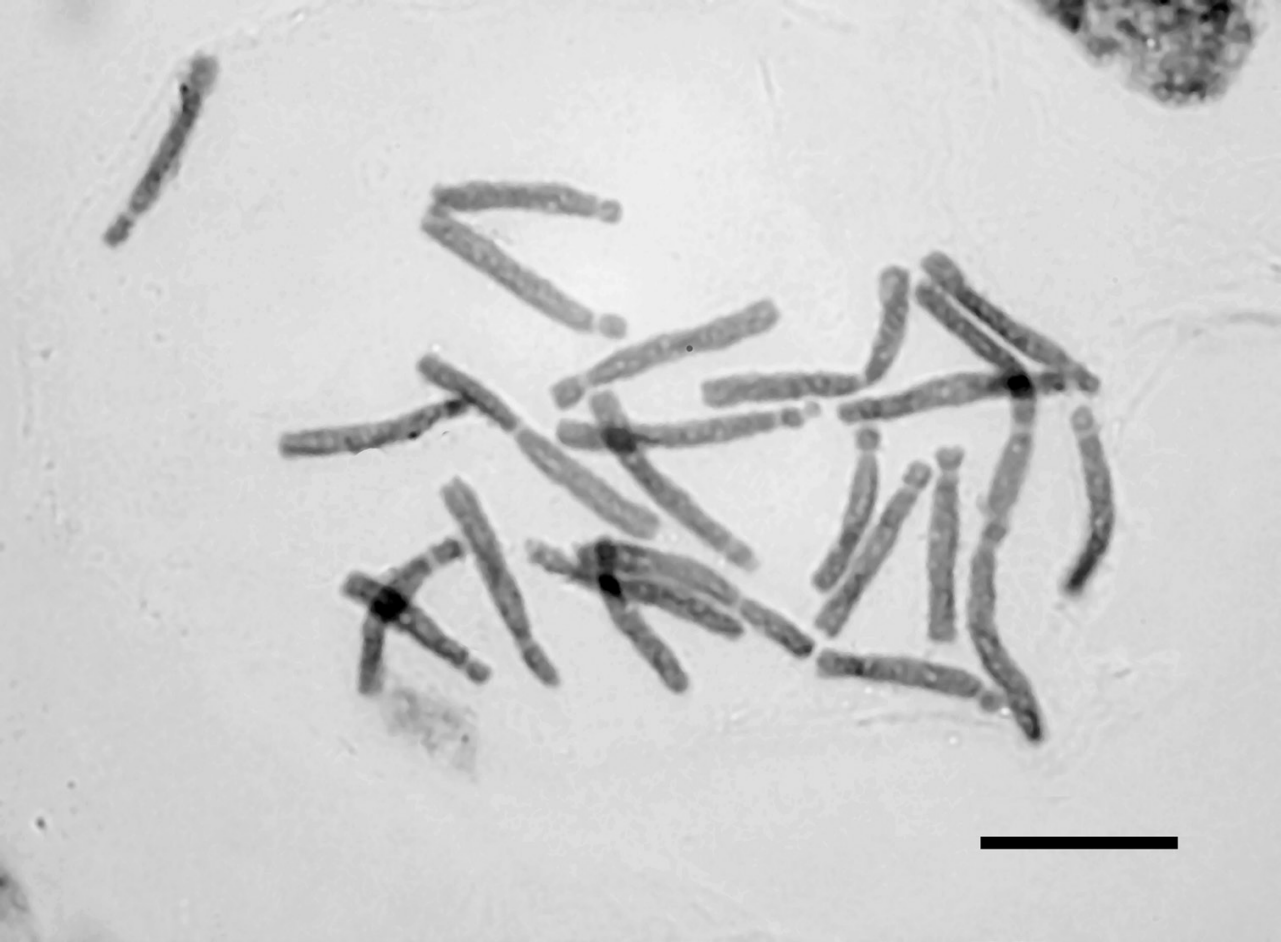
Photomicrograph of mitotic metaphase plate of *Fritillaria epirotica* from Mt. Smolikas, 2n = 2x = 24. Bar = 10 µm.

**Figure 6.**
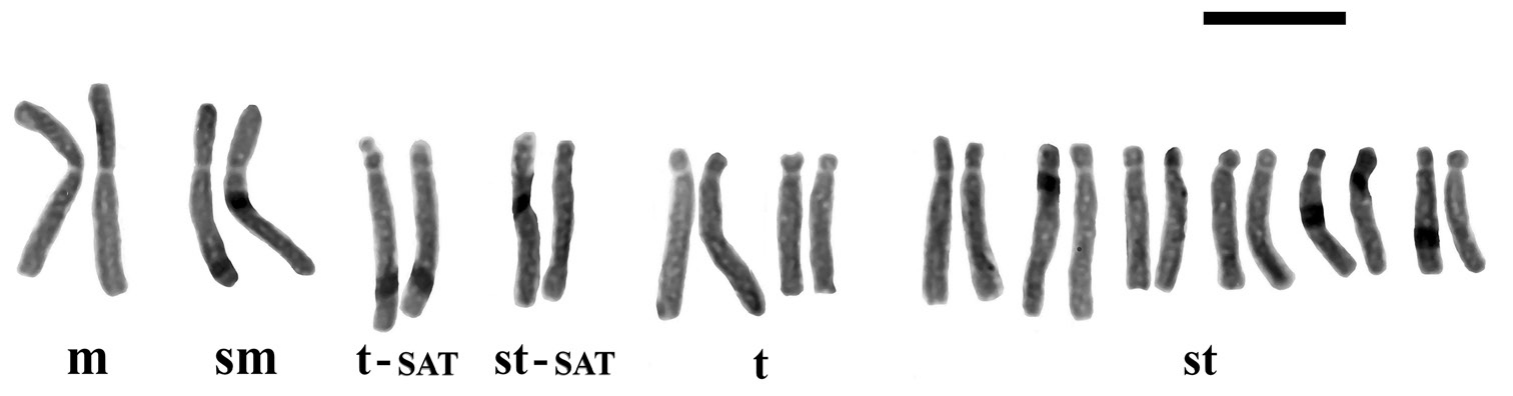
Karyogram of *Fritillaria epirotica* from Mt. Smolikas, 2n = 2x = 24. Bar =10 µm.

***Fritillaria epirotica*** Turrill ex Rix — 2n = 2x = 24 (Figs 5, 6).

Populations karyologically studied:

**Greece: Epirus: Nomos Ioanninon**: Katara Pass, prope ekchionistikos stathmos, alt. 1750 m, in apertis (*Pinus* Linnaeus, 1753; *Buxus* Linnaeus, 1753 etc), solo serpentinico, 4 May 1990, D. Phitos & G. Kamari 21348 (UPA); Eparchia Metsovou, Katara Pass, close to the second snowplow station, c. 13.5 km of Metsovon along the road to Trikala, slopes with *Pinus nigra* Arnold, 1785 and *Buxus sempervirens* Linnaeus,1753; ophiolithic substrate, alt. c. 1640 m, 39°47'N; 21°13'E, 24 Jun 1998, Th. Constantinidis 7919 (UPA); **Macedonia: Nomos Grevenon**: Mt. Vasilitsa, alt. 1764 m, 17 May 2015, G. Kofinas s.n. (cult. no. SF76, ACA); Mt. Smolikas, alt. 2200 m, Aug 2015, G. Kofinas s.n. (cult. no. SF97, ACA). **Thessalia: Nomos Trikalon**: Ep. Kalampakas, Mt. Chasia (Kratsovo), stony slopes close to a forest road, c. 3.0–3.5 km from Kakoplevri village, serpentine, alt. c. 1100–1180 m, 39°48'N; 21°24'E, 15 Jun 2000, D. Phitos, G. Kamari & Th. Constantinidis s.n. (cult. no. 235, UPA); Ep. Kalampakas, Mt. Chasia (Mt. Kratsovon), c. 3.1 km WNW of Kakoplevri village on the foothills of the mountain, hills with low *Buxus sempervirens* and *Juniperus oxycendrus* Linnaeus,1753; serpentine substrate, alt. 1120–1160 m, 39°49'N; 21°24'E, 24 Jul 2006, Th. Constantinidis s.n. (cult. no. 235, UPA).

Unlike *Fritillaria montana*, *Fritillaria epirotica* has the same basic somatic number as the rest of the Greek *Fritillaria* taxa, x = 12. The karyotype consists of 2n = 2m + 2sm + 14st + 6t = 24 chromosomes (Figs 5, 6), which range in size between 18.44 and 10 µm, while the TCL is 324.39 µm (Table 2). The index for interchromosomal asymmetry is small (CV_CL_ = 16.85) contradicting the big intrachromosomal one (M_CA_ = 63.33), while the centromere position heterogeneity is 51.66. Satellites on the short arms of one telocentric (t) and one acrocentric (st) pair of chromosomes (Table 3, chromosome pairs no. 3 and no. 5) are observed. However, in most metaphase plates, three of them are usually visible.

**Table 3.** Karyomorphometric indices of marker chromosomes for each species, marker chromosome pairs (numbered according to their chromosome length), long arm’s length (l), short arm’s length (s), chromosome length (l + s) with minimum and maximum prices, r- index, Centromeric index, Arm difference ratio, R-length.

Species	*Fritillaria montana*		*Fritillaria epirotica*			
Chromosome number	2n = 18		2n = 24			
marker chromosomes	Pair no. 1	Pair no. 5	Pair no. 1	Pair no. 2	Pair no. 3	Pair no. 5
**l (µm)** **(SD)**	12.84 (1.00)	10.71 (1.09)	9.68 (1.05)	10.51 (1.04)	12.19 (1.07)	10.87 (1.23)
**s (µm)** **(SD)**	11.15 (0.79)	6.93 (0.67)	6.86 (0.82)	5,27 (0.63)	1.60 (0.44)	1,82 (0.36)
**l + s (µm)** **(SD)**	23.99 (1.71)	17.65 (1.66)	16.53 (1.73)	15.77 (1.49)	13.79 (1.18)	12.42 (1.51)
**min l + s (µm)**	20.88	15	13.53	12.94	11.47	8.40
**max l + s (µm)**	26.47	20	19.12	18.24	15.59	15
**r-index** **l/s**	1.15	1.54	1.40	2.03	8.06	6.24
**Centromeric index** **l/l + s**	0.54	0.61	0.58	0.67	0.88	0.85
**Arm difference ratio** **l - s/l + s**	0.70	0.21	0.17	0.34	0.77	0.71
**R-length** **l + s/Sn(l + s)**	0.08	0.06	0.05	0.05	0.04	0.04

According to paired t-tests made (Table 4), the two species display an interesting similarity regarding their total chromosome length, but as far as the interchromosomal and intrachromosomal asymmetries are concerned (Table 5), the species seem to be clearly distinct. The only insignificant difference was revealed between the two cytotypes of *Fritillaria montana*, 2n = 18 and 2n = 27, as expected, since they both bear a lot of metacentric chromosomes (by Robersonian fusions).

**Table 4.** Paired t-tests between the three species regarding the TCL and ACL, along with degrees of freedom (df) and Significance (Sig) for every parameter. Bold characters are used for P values (Sig 2-tailed) under 0.01, which reveal significant statistical difference.

Species in comparison		TCL			ACL		
		t	df	Sig (2-tailed)	t	df	Sig (2-tailed)
*Fritillaria epirotica* 2n = 2x = 24	*Fritillaria montana* 2n = 2x =18	0.379	18	0.709	-4.347	18	**0.000**
*Fritillaria epirotica* 2n = 2x = 24	*Fritillaria montana* 2n = 3x = 27	-1.427	16	0.173	0.057	16	0.955
*Fritillaria montana* 2n = 2x = 18	*Fritillaria montana* 2n = 3x = 27	-1.947	10	0.080	3.898	10	**0.003**

**Table 5.** Paired t-tests between the three species regarding the CV_CL_ and M_CA_, along with degrees of freedom (df) and Significance (Sig) for every parameter. Bold characters are used for P (Sig 2-tailed) under 0.01, which reveal significant statistical difference.

Species in comparison		CV_CL_			M_CA_		
		t	df	Sig (2-tailed)	t	df	Sig (2-tailed)
*Fritillaria epirotica* 2n = 2x = 24	*Fritillaria montana* 2n = 2x = 18	-8.598	18	**0.000**	44.847	18	**0.000**
*Fritillaria epirotica* 2n = 2x = 24	*Fritillaria montana* 2n = 3x = 27	-9.754	16	**0.000**	34.473	16	**0.000**
*Fritillaria montana* 2n = 2x = 18	*Fritillaria montana* 2n = 3x = 27	-4.066	10	**0.002**	1.995	10	0.074

## Discussion

In the present study a detailed karyomorphological analysis of *Fritillaria montana* and *Fritillaria epirotica*, in material from Greece, was implemented focusing specifically to the study of the inter- and intrachromosomal asymmetry, as well as the detailed analysis of the marker chromosomes.

The study of marker chromosomes (Table 3) is always important since it can provide further information concerning genome organization and the differentiation of the karyotype between related species. Moreover, especially in the case of the genus *Fritillaria*, marker chromosomes are helpful for the distinction of the chromosome homologues, which is very difficult since the karyotype usually consists of mostly acrocentric and subtelocentric chromosomes with similar size.

Marker chromosomes were observed in both *Fritillaria epirotica* with 2n = 2x = 24 and *Fritillaria montana* with 2n = 2x = 18 chromosomes. However, when it comes to triploid karyotypes of the same species, the secondary constrictions are not stable in number and position.

*Fritillaria epirotica* (2n = 24) has four marker chromosome pairs (Fig. 6). The first two chromosome pairs, which are the longest ones of the complement, have a different morphology than all the other chromosomes of the karyotype, which are acrocentric (st) and subtelocentric (t). The longest chromosome pair is metacentric (m) (no. 1), while the second one is the second in range of length and a submetacentric (sm) one (no. 2). The third marker chromosome pair (no. 3) is telocentric and bears small spherical satellite on the short arm of the homologues. Finally, the last marker chromosome pair is the fifth in length, comprising of two acrocentric satellited (st-SAT) chromosomes. The results are in agreement with previous studies by Kamari (1991a). Zaharof (1989) reported the heteromorphism of satellites’ length in one out of two SAT-chromosome pairs.

*Fritillaria montana* (2n = 18) has two marker chromosome pairs with secondary constrictions. The karyotype formula given here (2n = 10m + 2st + 6t = 18) differs from the previously reported karyotype of 2n = 10m + 8t = 18 chromosomes given by Zaharof (1989). This is the only one species in Greece with 18 chromosomes and this chromosomal reduction has already been claimed as the result of successive chromosomal reconstructions and Robersonian-fusion of six acrocentric chromosomes into three metacentric ones (Darlington 1936, La Cour 1978a, 1978b, 1978c, Kamari 1991a). Zaharof (1989) explained the secondary constrictions, which are also observed in the present study, with the above hypothesis. Recently, Peruzzi et al. (2016) studied an Italian population with 2n = 2x = 18 chromosomes, further confirming the chromosome number of *Fritillaria montana*, while the presence of up to three B-chromosomes is already referred by Kamari (1991a, 1991b).

The triploid chromosome number of *Fritillaria montana* (2n = 3x = 27) is known in Greece from only one population, but it has also been reported from Italy by Cesca (1986, under the name *Fritillaria tenella* Marschall von Bieberstein, 1808), for a Calabrian population (S Italy).

Paired t-tests have revealed similarities among the three karyotypes. Especially the similarity between TCL of the diploid *Fritillaria epirotica* 2n = 24 and *Fritillaria montana* 2*n* = 18 reinforces the hypothesis, apart from the secondary constrictions, that the second species has derived after successive chromosomal reconstructions and Robersonian-fusions. Less similar indices of TCL between *Fritillaria montana* 2n = 2x = 18 and *Fritillaria montana* 2n = 3x = 27 can also be explained since it is known that polyploidy usually comes with gene loss and genome amount reduction (Kamari 1992, Leitch and Bennett 2004, Adams and Wendel 2005, Buggs et al. 2009). Another proof for gene loss, is the fact that the triploid cytotype of *Fritillaria montana* has the lower price of THL.

The results concerning the heterogeneity of centromere position CV_CI_ and the intrachromosomal asymmetry M_CA_ are nothing but expected. Following the explanation of this index by Zuo and Yuan (2011), the higher price of CV_CI_ found here, belongs to *Fritillaria montana*, because the karyotype comprises of mostly metacentric chromosomes. On the contrary, the higher price of M_CA_ belongs to *Fritillaria epirotica*, as it has a typical asymmetrical karyotype according to Stebbins (1971).

**Figure 7.**
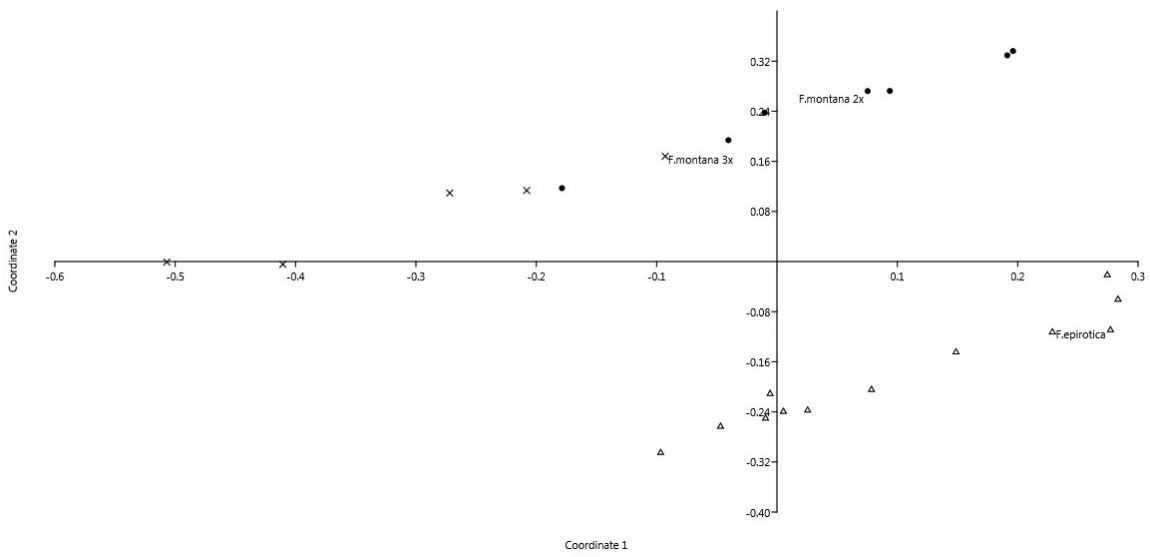
PCoA analysis based on six quantitative karyologial parameters. Triangle depicts *Fritillaria epirotica*, 2n = 2x = 24; dots *Fritillaria montana*, 2n = 2x = 18 and x *Fritillaria montana*, 2n = 3x = 27.

In total, the multivariate analysis PCoA confirms all above findings. More precisely, it presents all the accessions belonging to the same species close to each other. The two cytotypes of *Fritillaria montana* overlap, while the two species are clearly separated (Fig. 7).

Generally, karyological characteristics, as chromosome number, ploidy level, centromere position, and the number and location of satellites and secondary constrictions, can be used in elucidating taxonomical relationships of several plant taxa (Bareka et al. 2008, 2012 see for references). Although, karyomorphometrics is able to provide more information about the studied taxa, the conclusions can be used only as additional evidences to the primary hypothesis. However, molecular chromosomal markers and fluorescence in situ hybridization (FISH) could provide additional information concerning genome organization in the genus and differentiation among its species and are recommended as a safer way to reveal whether our assumption for the origin of the reduced chromosome number 2n = 18 is correct. Moreover, this method is desirable to be carried out because it will unveil the type of polyploidy for 2n = 3x = 27, as an autopolyploidy or allopolyploidy (Bareka et al. 2012).
